# Peripheral T-Cell Lymphoma of the Submandibular Salivary Gland as an Unusual Manifestation of Richter's Syndrome: A Case Report and Literature Review

**DOI:** 10.1155/2017/1262368

**Published:** 2017-12-03

**Authors:** Vadim R. Gorodetskiy, Natalya A. Probatova, Tatiana T. Kondratieva

**Affiliations:** ^1^Department of Intensive Methods of Therapy, V.A. Nasonova Research Institute of Rheumatology, Russian Academy of Medical Sciences, Kashirskoye shosse 34A, Moscow 115522, Russia; ^2^Department of Pathology, N.N. Blokhin Russian Cancer Research Center, Russian Academy of Medical Sciences, Kashirskoye shosse 24, Moscow 115478, Russia

## Abstract

Richter's syndrome is the development of high-grade non-Hodgkin lymphoma (NHL) or Hodgkin lymphoma in patients with chronic lymphocytic leukemia (CLL)/small lymphocytic lymphoma (SLL). In most patients with Richter's syndrome, the high-grade NHL is diffuse large B-cell lymphoma. Only a small minority of CLL/SLL patients develop T-cell malignancies. Herein, we describe a 40-year-old male patient presenting with peripheral T-cell lymphoma not otherwise specified (PTCL-NOS) in the submandibular salivary gland, two years after the diagnosis of CLL/SLL. The PTCL-NOS consisted of small lymphocytes, which complicated diagnosis. Immunohistochemical, cytological, and molecular studies allowed the correct diagnosis of composite lymphoma (SLL/PTCL-NOS) of the submandibular salivary gland. The PTCL-NOS had a cytotoxic phenotype and aberrant expression of CD79a. There was no evidence to suggest that the PTCL-NOS of the submandibular salivary gland developed from an intimately associated submandibular lymph node or by PTCL-NOS dissemination. A review of the literature and presented case suppose that the PTCLs developed following CLL/SLL have the cytotoxic phenotype and can clinically mimic typical Richter's syndrome.

## 1. Introduction

Richter's syndrome is the development of high-grade non-Hodgkin lymphoma (NHL) or Hodgkin lymphoma in patients with chronic lymphocytic leukemia (CLL)/small lymphocytic lymphoma (SLL) [1]. In most patients with Richter's syndrome, the high-grade NHL is diffuse large B-cell lymphoma. In rare cases, T-cell malignancies develop in patients with CLL/SLL. Single cases have been described of the combination of CLL/SLL and anaplastic large-cell lymphoma (ALCL) [2, 3], T-cell large granular leukemia [4, 5], subcutaneous panniculitis-like T-cell lymphoma [6], cutaneous gamma-delta T-cell lymphoma [7], mycosis fungoides [8], and peripheral T-cell lymphoma, not otherwise specified (PTCL-NOS) [8–12]. As some of the cases have been reported a while back [13–17], not all the peripheral T-cell lymphomas (PTCLs) have been subclassified as per the most recent World Health Organization Classification [18]. Herein, we describe an unusual manifestation of Richter's syndrome in the form of extranodal PTCL-NOS in the submandibular salivary gland.

## 2. Case Presentation

**Figure 1:**
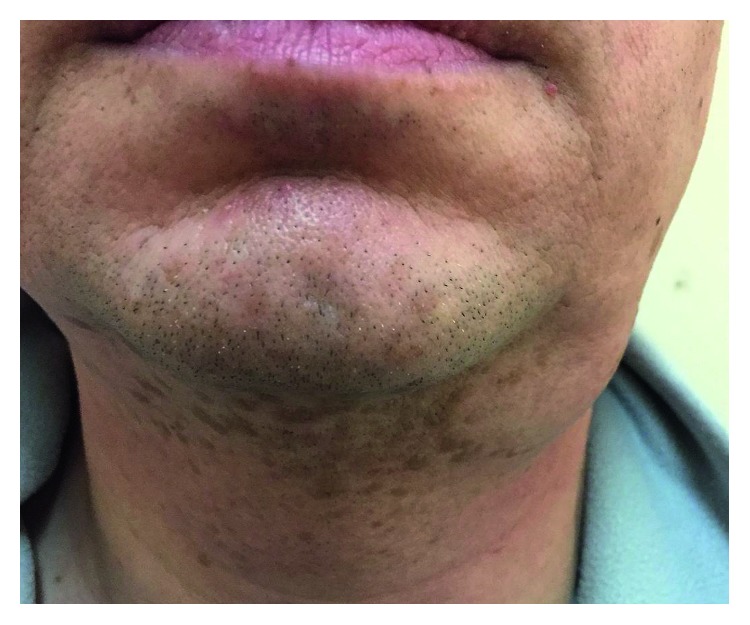
A mass in the left submandibular region.

**Figure 2:**
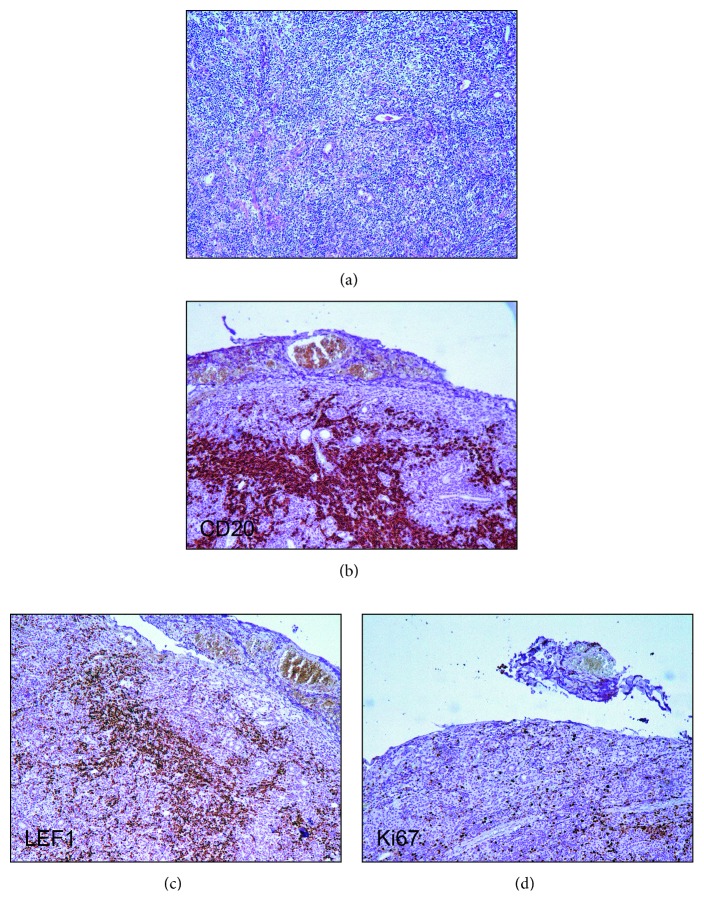
Submandibular salivary gland. (a) The architecture of the submandibular salivary gland is replaced by a diffuse small lymphocyte infiltrate H&E staining, ×100. (b) The SLL component strongly expresses CD20, highlighting focal accumulations of CD20+ B cells, ×100. (c) B cells express LEF1, ×100. (d) Proliferative activity marker Ki-67 in the B-cell area is expressed by up to 20% of the cells, ×100.

**Figure 3:**
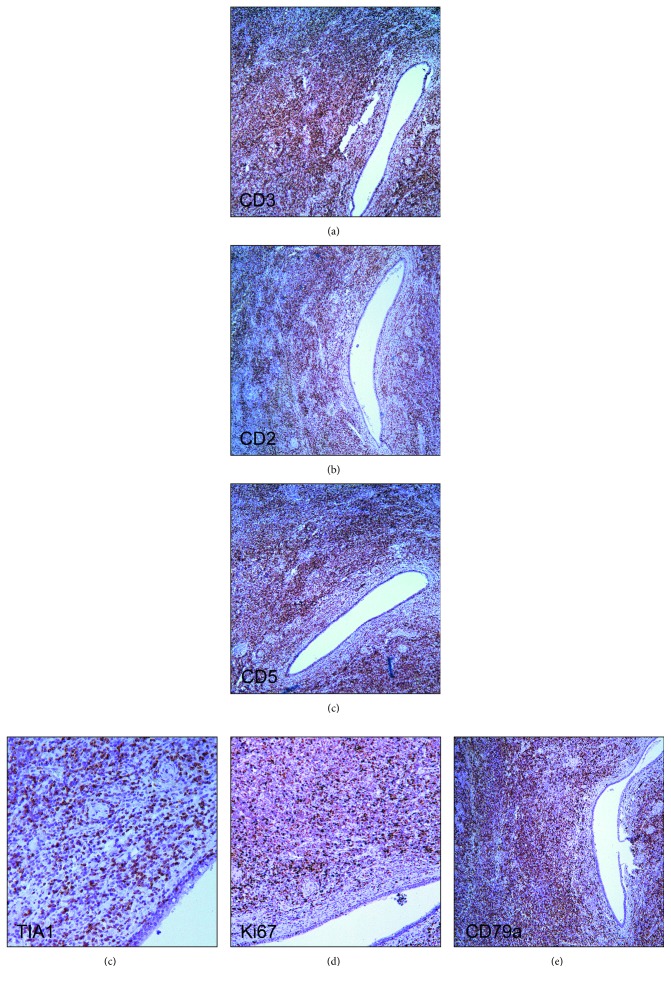
Submandibular salivary gland. (a) Immunohistochemistry for CD3 shows diffuse infiltration by CD3+ T cells, ×50. (b) T cells express CD2, ×50. (c) T cells express CD5, ×50. (d) T cells express TIA1, ×200. (e) Proliferative activity marker Ki-67 in the T-cell area is expressed by up to 70% of the cells, ×100. (f) The neoplastic T cells coexpress CD79a, ×50.

**Figure 4:**
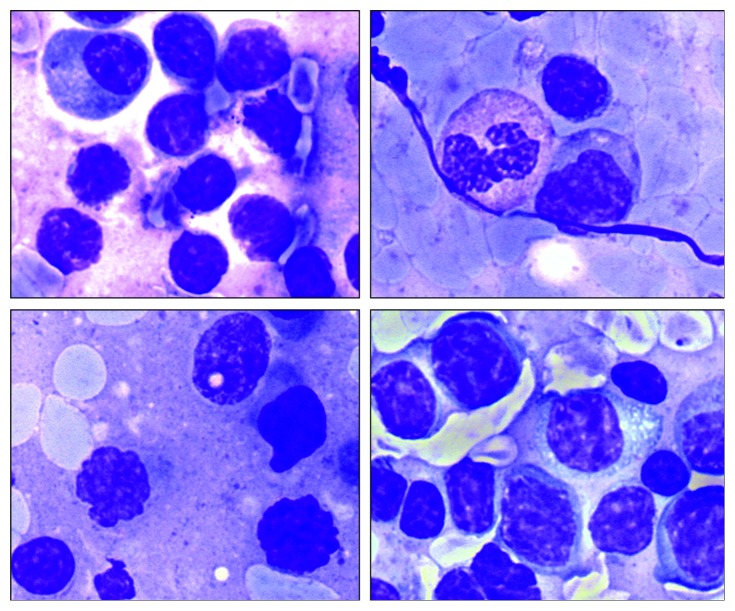
The cytology of the imprint of the submandibular salivary gland. Two lymphocyte populations can be detected. Some lymphocytes correspond to the characteristics of CLL/SLL lymphocytes. The others have an irregular shape of the nucleus, and some contain cytoplasmic granules. Romanowsky–Giemsa staining, ×1000.

**Figure 5:**
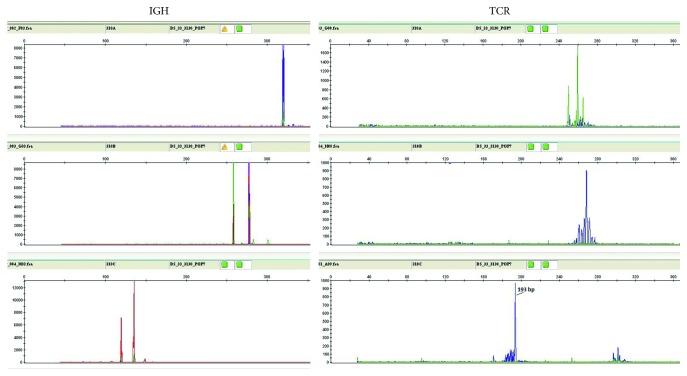
Molecular diagnostics of composite lymphoma. Clonality of the B cells and T cells was assessed for IGH and TCRβ gene rearrangements by PCR. Clonal peaks of IGH rearrangement were detected with framework I–III primers. An incomplete rearrangement was revealed of β chain of T-cell receptor: a clonal peak at 193 bp (panel C).

**Figure 6:**
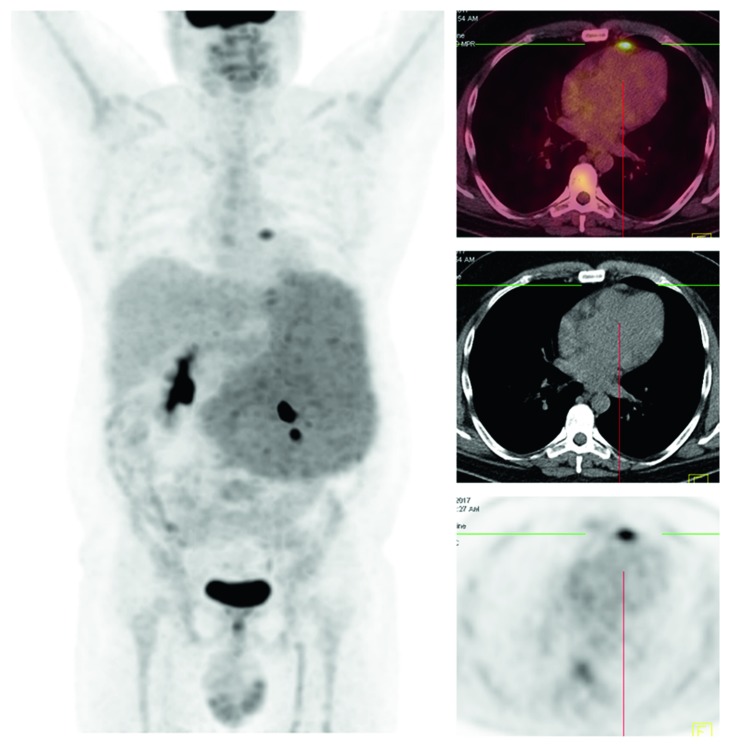
Positron emission tomography-computed tomography. Enlarged pericardial lymph node in the anterior mediastinum with SUVmax = 6.1.

A previously healthy 38-year-old man presented with enlarged lymph nodes in the left supraclavicular and both axillary and inguinal areas. Computed tomography also revealed an increase of mediastinal, hilar, abdominal, retroperitoneal, retrocrural, and iliac lymph nodes, as well as hepatosplenomegaly. Peripheral blood flow cytometry showed 2095 B cells/mL with a CLL-like immunophenotype: CD19+, CD5+, CD23+, CD38+, CD79b−/CD43+, and CD22low/CD81low. Histology of an enlarged right axillary lymph node showed a distortion of the lymph node architecture by small lymphocytes expressing CD20, CD5, CD23, CD43, BCL2, and LEF1, consistent with SLL involvement. Peripheral blood mononuclear cells analyzed by FISH showed ATM (ataxia-telangiectasia mutated) gene deletion (11q23) in 25% of nuclei. A diagnosis of CLL/SLL stage II/B (Rai/Binet) was made. During the next two years, the disease remained stable and did not require any therapy. During a monitoring visit in March 2017, the patient noted the appearance of a dense painless mass in the left submandibular area, which rapidly increased in size within a month (Figure 1). The serum lactate dehydrogenase (LDH) level was elevated to 368 IU/L (normal < 225). Richter's syndrome was suspected. Ultrasound scanning and computed tomography did not allow categorical target organ identification (submandibular lymph node or submandibular salivary gland). Histologic examination revealed diffuse infiltration of the salivary gland tissue with small lymphocytes with atrophy of glandular parenchyma (Figure 2). Immunohistochemical staining showed that only a part of the infiltration, mainly the focal cluster, was composed of lymphocytes expressing CD20 (Figure 2), CD79a, PAX5, CD5, CD23, LEF1 (Figure 2), and CD43. Up to 20% cells of the focal cluster expressed the proliferative activity marker Ki-67 (Figure 2). The lymphoid infiltration consisted mostly of cells expressing CD2, CD3, CD5, CD7, CD8, CD43, TIA1, and granzyme B and coexpressing CD79a, with 70% Ki-67 positivity (Figure 3). The study of imprints of the submandibular salivary gland showed two types of lymphocytes: one corresponding to CLL/SLL lymphocytes and the other with irregular nuclei and cytoplasmic granules (Figure 4). Study of the DNA extracted from freshly prepared submandibular salivary gland tissue revealed clonal IGH and clonal TCRβ gene rearrangements (Figure 5). A composite SLL/PTCL-NOS lymphoma of the submandibular salivary gland was diagnosed. Positron emission tomography-computed tomography showed an increase in the size of the spleen, right submandibular, bilateral cervical, left supraclavicular and axillary, mediastinal, retrocrural, abdominal, retroperitoneal, and bilateral external iliac lymph nodes, with slightly increased accumulation of fluorine-18 fluorodeoxyglucose (SUV (standard uptake value) max = 3.7) as well as an enlarged pericardial lymph node in the anterior mediastinum (SUVmax = 6.1) (Figure 6). The patient refused a diagnostic biopsy of the pericardial lymph node, bone marrow study, and therapy. Seven months after the diagnosis of extranodal PTCL-NOS, the patient feels well.

## 3. Discussion

Composite or discordant T- and B-cell lymphomas are uncommon, and T-cell lymphomas associated with CLL/SLL have been only rarely reported. A review of the literature shows 11 cases of CLL/SLL with PTCL-NOS (Table 1). In 6 cases, lymphomas were discordant (CLL/SLL and PTCL-NOS occurrence in separate anatomic sites), and in 5 cases, composite ones (CLL/SLL and PTCL-NOS occurrence in the same anatomical localization). In 4 cases, composite lymphoma was verified by biopsy of the lymph node, and in one case, by bone marrow biopsy. In all cases, CLL/SLL preceded PTCL-NOS, or both lymphomas were diagnosed simultaneously. In our case, SLL preceded the development of PTCL-NOS by 2 years. The rapid growth of an isolated mass in the submandibular region and increase of LDH suggested the development of Richter's syndrome. Histology revealed that the lesion was localized in the submandibular salivary gland. Diffuse infiltration of submandibular salivary gland tissue by small lymphocytes initially suggested SLL origin. Unlike other reports that have described a composite SLL and PTCL-NOS lymphoma [8, 9, 11], in our case, PTCL consisted of small lymphocytes, which precluded histological verification. The discrepancy in the clinical picture (rapid growth of the tumor) and the unspecific histological picture prompted us to conduct further studies. Immunohistochemistry showed a cytotoxic (CD8+, TIA1+, and granzyme B+) T-cell infiltrate with high proliferative activity (70% Ki-67 positivity), with infiltrating B-lymphocytes foci of CLL/SLL immunophenotype showing significantly less proliferative activity (20% Ki-67 positivity). Molecular analysis detected monoclonal rearrangement of the IGH and TCRβ genes in the infiltrating cells. Cytological study of imprints of the submandibular salivary gland revealed two different lymphoid populations. Thus, a combination of immunohistochemistry, molecular, and cytological studies allowed the diagnosis of composite SLL/PTCL-NOS lymphoma of the submandibular salivary gland.

The two lymphomas in the submandibular salivary gland were not clearly delineated, which made it difficult to assess their immunophenotype. Nevertheless, a careful evaluation of the immunohistochemical picture of the composite lymphoma uncovered the aberrant expression of CD79a by T cells of PTCL-NOS. Literature shows 7 cases of peripheral T-cell lymphomas with aberrant CD79a expression [19–22] (Table 2), and in all 6 cases where the expression of cytotoxic molecules was evaluated, tumor T cells had a cytotoxic phenotype. In addition, 5 out of 7 cases were extranodal lymphomas.

Salivary gland lymphomas account for approximately 5% of all extranodal lymphomas [23], 1% to 2% of all salivary gland neoplasms [24], and about 10% of all salivary gland malignancies [25]. Lymphomas of the salivary glands are biologically heterogeneous and inevitably include genuine extranodal lymphomas and nodal-type lymphomas, with the latter arising in intrasalivary lymph nodes [26]. It can be difficult to distinguish between the two types because the lymphoma cells often spill over into the adjacent tissues. The definition of a true primary salivary gland lymphoma is an issue. Schmid et al. reported that if a lesion has no capsule-like structure reminiscent of an expanded lymph node capsule and no lymph nodes, it can be defined as a true primary salivary gland lymphoma [27]. The majority of salivary gland lymphomas are of B-cell lineage. In contrast, primary salivary gland T-cell and NK-cell lymphomas are extremely rare. To the best of our knowledge, only 16 such cases have been reported [28–34]. Nine cases were parotid salivary gland lymphomas (one case with intrasalivary gland lymph nodal origin), 5 were submandibular salivary gland lymphomas, and 2 were sublingual salivary gland lymphomas. Histological subtypes were variable and included PTCL-NOS, ALCL, NK-cell lymphoma, and adult T-cell lymphoma/leukemia. In our case, there was no evidence to suggest that the PTCL-NOS of the submandibular salivary gland developed from an intimately associated submandibular lymph node or PTCL-NOS dissemination.

The pathogenesis of PTCL-NOS lymphoma arising in association with CLL/SLL is not well understood, and several hypotheses may be conceived. It is intriguing that all cases of PTCL-NOS reported in CLL/SLL patients expressed cytotoxic granule proteins (Table 1, except one case where data were unavailable), in contrast to T-cell lymphomas in the general population [35, 36]. In our case, the PTCL-NOS also expressed cytotoxic proteins: TIA1 and granzyme B. Cytotoxic T-cell lymphomas are often associated with chronic antigenic stimulation or chronic immunosuppression [37]. The decreased immunosurveillance accompanying CLL/SLL is well documented, and studies of cellular immunity in CLL/SLL patients have found reduced T-cell function with a paradoxical clonal expansion of CD8+ T cells [8] and increased circulating CD8+ T-cell counts [38]. Theoretically, this could increase the incidence of T-cell large granular leukemia in CLL/SLL patients, but only 2 cases of such an association are described in the literature [4, 5].

The second possibility is the exposure to chemotherapy for CLL/SLL that induces a secondary neoplasm. Information on CLL/SLL therapy before the development of PTCL-NOS was provided for only 7 of 11 patients (Table 1). Four patients received multiagent chemotherapy including fludarabine, one patient received experimental therapy with interleukin 4, and in 2 patients, therapy was not administered (since both lymphomas were diagnosed simultaneously). In our patient, before development of PTCL-NOS, the CLL/SLL therapy was not indicated [39], and we adhered to the wait-and-watch approach.

It is unusual that the patient is feeling well without therapy within seven months after diagnosis of extranodal PTCL. We believe that the total resection of submandibular salivary gland resulted in the complete removal of the primary extranodal T-cell lymphoma. The assumption is supported by the PET-CT. However, we cannot definitively interpret the enlarged pericardial lymph node in the anterior mediastinum with the SUVmax = 6.1. Such an SUV can be observed in the lymph node lesion by both SLL [40] and PTCL as well [41].

We believe this is the first reported case of composite lymphoma (SLL/PTCL-NOS) of the submandibular salivary gland. A feature of this case was the absence of histological signs, indicating the presence of a second lymphoma in the salivary gland initially affected by SLL. The PTCL-NOS consisted of small lymphocytes with a cytotoxic phenotype and aberrant CD79a expression.

## Figures and Tables

**Table 1:** Review of the literature for PTCL-NOS in CLL/SLL patients.

Authors, reference	Age^∗^/sex	Interval, CLL/SLL to PTCL-NOS (mo)	Therapy for CLL/SLL, yes (+)/no (−)	Expression of PTCL-NOS cytotoxic molecules		Discordant lymphoma	Composite lymphoma (localization)
				TIA1	Granzyme B		
Martinez et al. [8]	70/M	156	NA	ND	ND	+	—
Martinez et al. [8]	73/M	ND	NA	+	+	—	+ (LN)
Martinez et al. [8]	59/M	10	+ (PCT)	−	+	+	—
Martinez et al. [8]	73/M	60	+ (IL-4)	+	+	+	—
Martinez et al. [8]	57/M	168	NA	+	+	+	—
Martinez et al. [8]	58/F	ND	NA	+	+	—	+ (LN)
Campidelli et al. [9]	80/M	−	−	+	+	—	+ (LN)
Campidelli et al. [9]	61/M	−	−	ND	+	—	+ (BM)
Buddula and Assad [10]	66/M	72	+ (PCT)	+	+	+	—
Alomari et al. [11]	68/F	84	+ (PCT/RT)	−	+	—	+ (LN)
Boyer et al. [12]	70/M	132	+ (PCT)	ND	+	+	—

^∗^At PTCL-NOS diagnosis; LN: lymph node; BM: bone marrow; PCT: polychemotherapy; RT: radiotherapy; IL-4: interleukin 4; NA: not available; ND: not done; PTCL-NOS: peripheral T-cell lymphoma not otherwise specified; CLL/SLL: chronic lymphocytic leukemia/small lymphocytic lymphoma; TIA1: T-cell intracellular antigen-1.

**Table 2:** Review of the literature for peripheral T-cell lymphomas with aberrant CD79a expression.

Authors, reference	Age/sex	Lymphoma localization		Expression of cytotoxic molecules	
		Lymph node	Extranodal	TIA1	Granzyme B
Blakolmer et al. [19]	77/M	−	Nose	+	+
Blakolmer et al. [19]	63/M	−	Jejunum	+	+
Blakolmer et al. [19]	42/M	−	Jejunum	+	+
Blakolmer et al. [19]	45/M	−	Stomach	+	+
Yao et al. [20]	76/M	−	Left adrenal gland	+	ND
Bo et al. [21]	77/M	+	−	+	+
Matnani et al. [22]	75/M	+	−	ND	ND

ND: not done; TIA1: T-cell intracellular antigen-1.

## Data Access

The datasets used and/or analyzed during the current study are available from the corresponding author on reasonable request.
